# Phosphorylation of NMDA receptors by cyclin B/CDK1 modulates calcium dynamics and mitosis

**DOI:** 10.1038/s42003-020-01393-3

**Published:** 2020-11-12

**Authors:** Margarita Jacaranda Rosendo-Pineda, Juan Jesus Vicente, Oscar Vivas, Jonathan Pacheco, Arlet Loza-Huerta, Alicia Sampieri, Linda Wordeman, Claudia Moreno, Luis Vaca

**Affiliations:** 1 Instituto de Fisiología Celular, Universidad Nacional Autónoma de México, Ciudad Universitaria, Coyoacán, DF 04510 Mexico; 2 Department of Physiology and Biophysics, University of Washington School of Medicine, Seattle, WA 98195 USA

**Keywords:** Mitosis, Molecular biophysics, Chromosome segregation

## Abstract

N-methyl-D-aspartate receptors (NMDAR) are glutamate-gated calcium channels named after their artificial agonist. NMDAR are implicated in cell proliferation under normal and pathophysiological conditions. However, the role of NMDAR during mitosis has not yet been explored in individual cells. We found that neurotransmitter-evoked calcium entry via endogenous NMDAR in cortical astrocytes was transient during mitosis. The same occurred in HEK293 cells transfected with the NR1/NR2A subunits of NMDAR. This transient calcium entry during mitosis was due to phosphorylation of the first intracellular loop of NMDAR (S584 of NR1 and S580 of NR2A) by cyclin B/CDK1. Expression of phosphomimetic mutants resulted in transient calcium influx and enhanced NMDAR inactivation independent of the cell cycle phase. Phosphomimetic mutants increased entry of calcium in interphase and generated several alterations during mitosis: increased mitotic index, increased number of cells with lagging chromosomes and fragmentation of pericentriolar material. In summary, by controlling cytosolic calcium, NMDAR modulate mitosis and probably cell differentiation/proliferation. Our results suggest that phosphorylation of NMDAR by cyclin B/CDK1 during mitosis is required to preserve mitotic fidelity. Altering the modulation of the NMDAR by cyclin B/CDK1 may conduct to aneuploidy and cancer.

## Abstract (web summary)

Rosendo-Pineda et al. discover that N-methyl-D-aspartate receptors (NMDAR) mediate transient calcium entry in mitosis, dependent on phosphorylation of NMDAR by Cyclin B1/CDK1. They also find that phosphorylation-mimicking mutants of NMDAR cause mitotic defects, suggesting that precise control of NMDAR-mediated calcium entry is needed for proper mitosis.

## Introduction

NMDAR are ion channels activated by the binding of neurotransmitters glutamate or aspartate, or by artificial ligands like NMDA. These receptor-channels permeate cations including calcium ions and are assembled as heterotetramers from any of the seven members of the NMDAR family. Three subtypes of NMDAR have been identified: NR1, NR2 which divides into 2A, 2B, 2C and 2D, and NR3 which divides into 3A and 3B^1,2^. NR1 is required to form functional channels in mammalian cells^3–5^. NMDAR have critical roles in the central nervous system function and are involved in neuropathological disorders^3^. In the central nervous system, these receptor-channels are expressed in both neurons and glial cells such as astrocytes. NMDAR participate in multiple processes including neural plasticity, memory and learning^6^. However, in various neuropathological conditions when overactivated and by mediating large amounts of calcium into the cytosol, these receptors lead to excitotoxic cell death; in contrast, NMDAR promote schizophrenia when under-activated^7–9^. Under normal and pathophysiological conditions, NMDAR can play roles in proliferation. Thus, they control proliferation and differentiation of hippocampal neural progenitor cells^10^, and participate in the invasion and proliferation of glioblastoma cells^11,12^. Unlike fully differentiated neurons, astrocytes can initiate cell cycles and undergo cell division.

The eukaryotic cell cycle alternates between two stages: interphase and mitosis. During interphase, cells grow (G1 phase), duplicate their genetic material (S phase) and synthesize all the proteins required for mitosis (G2 phase). During mitosis, cells divide and segregate their genetic material into the two daughter cells. This process is highly regulated to assure that the transmission of genetic information is efficient and error-free^13^. The appearance of lagging chromosomes during anaphase is a common readout for unresolved mitotic errors. If errors occur during chromosome segregation the daughter cells complete mitosis with differing amounts of genetic material and/or variations in chromosome number, a condition called “aneuploidy”^14,15^. Aneuploidy is frequently observed in cancer cells, leading to the hypothesis that errors in mitosis can generate cancerous phenotypes^16,17^. For this reason, mitosis in all living organisms is highly regulated to minimize mitotic errors^18,19^. The key proteins that regulate cell cycle progression are cyclins and cyclin-dependent kinases (CDK). Cyclins bind CDK and form cyclin/CDK complexes that initiate the kinase activity of the CDK, and regulate the transitions between different phases of the cell cycle^20,21^.

The formation of the cyclin B/CDK1 complex initiates G2/M transition, and exit from mitosis requires the disassembly of the cyclin B/CDK1 complex during the metaphase–anaphase transition^22^. CAMKinase II and calcium ions participate in checkpoints during G2-M and the metaphase–anaphase, therefore, regulation of the cytosolic calcium concentration ([Ca^2+^]_i_) is crucial for the accurate start and end of mitosis^23^. [Ca^2+^]_i_ increase is required for CDK1 to enter to the nucleus, and calcium–calmodulin triggers the phosphorylation of CDK1 and promotes cyclin B ubiquitination, signaling the end of mitosis. Calcium also activates chromosome-associated Topoisomerase II, which enables the sister chromatids to separate^24^. It has been shown that calcium and other ions such as magnesium fluctuate throughout the cell cycle, strongly suggesting an interrelation between ion channels activity and progress through the cell cycle^25–27^. While there is some evidence for cell cycle-dependent modulation of ion channels, considerably less evidence exists about the mechanism by which ion channels regulate cell cycle progression^28,29^.

Ion channels have also been implicated in signaling roles related to mitosis, such as interactions with cyclins/CDK and phosphatases and regulation of [Ca^2+^]_i_^30–34^. There are reports that blocking of voltage-gated Ca_V_1.2 and Ca_V_1.3 channels in neuroendocrine cells facilitates their arrest in mitosis, and phosphorylation of STIM1 by CDK1 decrease calcium entry through Orai channels during mitosis^35,36^. NMDAR activity has been associated with proliferation of glioblastoma cells^12^, and with proliferation and differentiation of hippocampal neural progenitor cells^10^. Still, the molecular mechanisms linking NMDAR modulation to cell cycle, particularly mitosis, remain unknown. In the present study, we show evidence that the cyclin B/CDK1 complex phosphorylates NMDAR during mitosis. NMDAR phosphorylation reduces activity-dependent run-down of these receptors and promotes persistent calcium entry. We propose that this persistent calcium entry leads to altered mitotic index, formation of multipericentric foci and lagging chromosomes in cells expressing phosphomimetic mutants, that recapitulate phosphorylated NMDAR by CDK1. The results presented here show the selective phosphorylation of the NMDAR during mitosis by cyclin B/CDK1 complex resulting in the modulation of channel activity to preserve mitotic fidelity.

## Results

### Calcium entry via NMDAR is reduced during mitosis in astrocytes and HEK293 cells

**Fig. 1:**
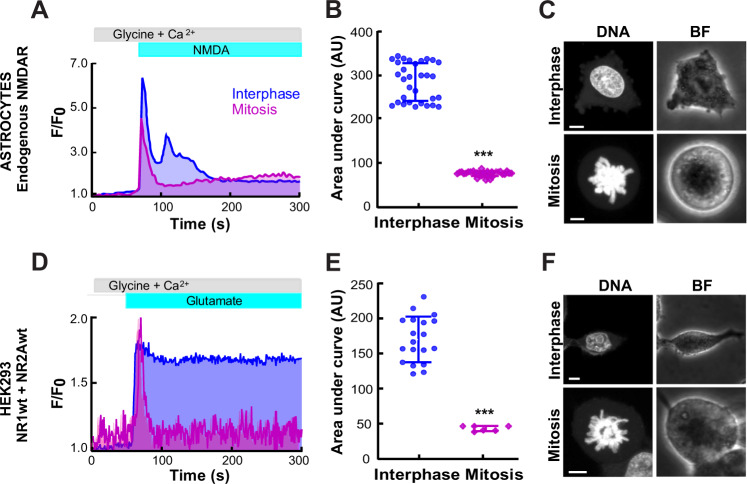
Calcium entry via NMDA receptors is reduced in mitotic astrocytes and mitotic HEK293 cells. **a** Representative traces of cytosolic calcium measurements in rat astrocytes for 300 s using Fura Red. Pink trace: a mitotic astrocyte. Blue trace: an astrocyte in interphase. The upper bars indicate the time when the glutamate, glycine and calcium ion were applied. **b** Summary plot of area under the curve (arbitrary units, AU) from the calcium addition to astrocytes comparing interphase (*n* = 32 cells) and mitotic astrocytes (*n* = 31 cells). **c** Confocal images of an interphase astrocyte (above) and a mitotic astrocyte (below). Left: DNA stain with Syto-59. Right: bright field (BF). **d** Representative traces of cytosolic calcium measurements in HEK293 cells transfected with wild-type NR1 and NR2A subunits of NMDAR. **e** Summary plot of area under the curve from the calcium addition to HEK293 cells comparing interphase (*n* = 19 cells) and mitotic HEK293 cells (*n* = 6 cells). **f** Confocal images of HEK293 cells. NMDAR-subunits were identified by the florescence of GFP. The scale bar on all confocal images is 5 µm. Data represent mean ± standard deviation with significance set at ***p < 0.0001, analyzed by two-tailed Student’s *t*-test in **b** and **e**.

Using primary cultures of cortical astrocytes, we evoked calcium entry with the selective ionotropic agonist NMDA. We observed that calcium influx through the endogenous NMDAR became transient when cells were undergoing spontaneous mitosis, compared to cells in interphase (Fig. 1a–c). We identified mitotic astrocytes using a DNA dye and looked for cells with DNA condensation and alignment of chromosomes at the equator plate, by spherical cell morphology and by rupture of the nuclear membrane.

Since astrocytes express several NMDAR subunits, we decided to move to a more controlled environment to study this phenomenon without interference from endogenous subunits, so we expressed NR1 + NR2A subunits in HEK293 cells. This combination is the most abundant in glia and neurons from human cerebral cortex^37^ and in rat cortical astrocytes^38^. Interestingly, the phenotype observed with astrocytes was reproduced in the HEK293 cells undergoing mitosis (Fig. 1d–f). Mitotic HEK293 cells expressing a combination of wild-type NR1 + NR2A produced a transient calcium elevation when stimulated with glutamate, whereas cells in interphase produced a sustained elevation (Fig. 1d–f).

### NMDAR are phosphorylated by cyclin B/CDK1 complex

Cyclin B/CDK1 complex is the central regulator triggering mitosis. Ablation or inhibition of CDK1 results in cell arrest prior to mitosis followed by cell death. CDK1 is also known to be the main CDK active during mitosis^20^. Thus, CDK1 was an obvious candidate to explore as a possible mechanism for NMDAR modulation during mitosis. To date there are no reports of NMDAR modulation by cyclin B/CDK1, and no reports of putative NMDAR consensus phosphorylation sequences for CDK.

**Fig. 2:**
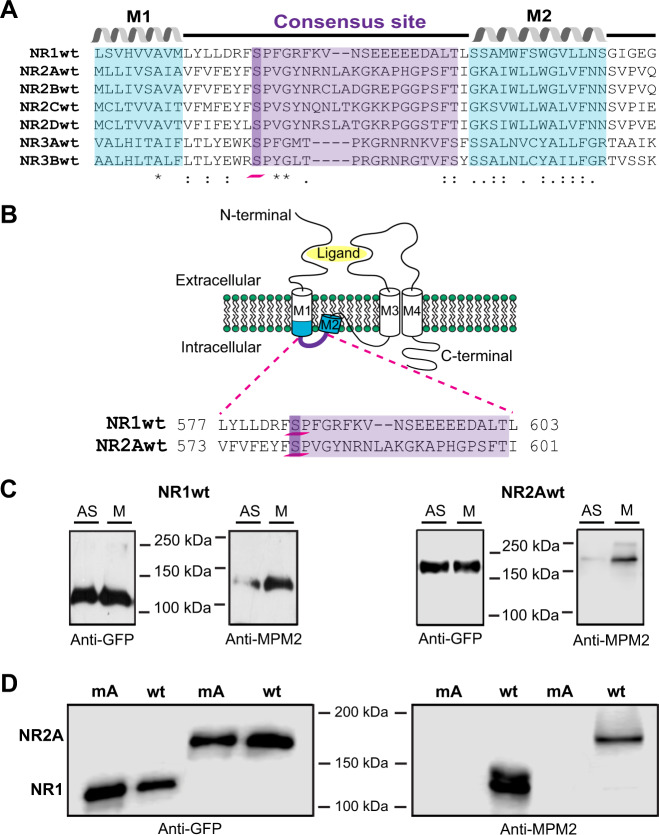
NMDA receptors are phosphorylated during mitosis. **a** A sequence alignment of all wild-type rat NMDAR-subunits showing a consensus site of phosphorylation by CDK. The consensus site of phosphorylation is highlighted in purple with the Serine that is phosphorylated by CDK highlighted in dark purple and underlined with a pink line. The amino acids of helix 2 (M2) and part of helix 1 (M1) are highlighted in blue. The alignment was performed with the Clustal W 2.0.12 software. **b** Topological diagram of NMDAR; cylinders represent alpha-helices and the purple line the intracellular loop that connects M1–M2 helices. The pink dotted lines indicate the residues that form the M1–M2 loop of the wild-type NR1 (top) and NR2A (bottom) subunits. Numbers before and after the sequences denote the position number in the whole sequence. Putative phosphorylation sites for CDK are serines S584 for NR1wt and S580 for NR2Awt (dark purple and underlined with a pink line). **c**) Western blots of NR1wt subunit (left) and NR2Awt subunit (right) immunoprecipitated from lysates of asynchronous-HEK293 cultures (AS) or arrested-cells in mitosis (M). First, immunoprecipitates were analyzed with the anti-GFP antibody to reveal total protein (NR1wt and NR2Awt), then, the blot was stripped and re-revealed with an anti-MPM2 antibody to detect total phosphorylated protein. Full scans of these blots are shown in Supplementary Fig. 1. **d** Western blots of immunoprecipitated from lysates of arrested-cells in mitosis and transfected with NMDAR-subunits mutated to prevent phosphorylation (alanine mutant, mA) and wild-type NMDAR-subunits (wt). Cells expressed combinations of NR1wt/NR2Awt, NR1mA/NR2Awt, and NR1wt/NR2AmA. The left blot shows total protein and the right blot shows phosphorylated protein.

However, an in silico analysis yielded a striking candidate: all NMDAR subunits possessed a unique putative consensus cyclin-dependent kinase phosphorylation site (Fig. 2a). This consensus site has not been previously reported among the plethora of phosphorylation sites for other kinases found at the N and C terminus of the NMDAR^39^. The consensus site lies at the first intracellular loop between the M1 and M2 transmembrane domains (Fig. 2b). To determine whether these sequences were indeed phosphorylated by a cyclin-dependent kinase we used the MPM2 monoclonal antibody, which recognizes phosphorylated S/TP motif and has been used to identify phosphorylated proteins by CDK1 during mitosis^36,40,41^. Inmunoprecipitation and western blot analysis showed that both NR1wt and NR2Awt are phosphorylated during mitosis but not in asynchronous cells, when comparing cells arrested in mitosis (M) with asynchronous (AS) cells (Fig. 2c, full length-blots in Supplementary Fig. 1). To demonstrate that the phosphorylation by CDK1 was at the serine present in the consensus site (Fig. 2b), we replaced serines 584 (NR1) and 580 (NR2A) with alanines. Replacing serine 584 (NR1) and 580 (NR2A) with alanines prevented the phosphorylation of both subunits from the NMDAR as reported by the lack of recognition by the MPM2 antibody (Fig. 2d, full-length blots in supplementary Fig. 2). As expected, we observed negligible phosphorylation of NR1 or NR2A during interphase (Fig. 2c). These results suggest that active CDK1 (cyclin B/CDK1 complex) is the kinase responsible for the phosphorylation of both subunits (S584 in NR1, and S580 in NR2A) from the NMDAR during mitosis.

Because several kinases are active during mitosis, and the MPM2 antibody does not differentiate among CDK, we decided to perform in vitro phosphorylation assays using a purified Cyclin B1/CDK1 complex (see “Materials and methods” section).

**Fig. 3:**
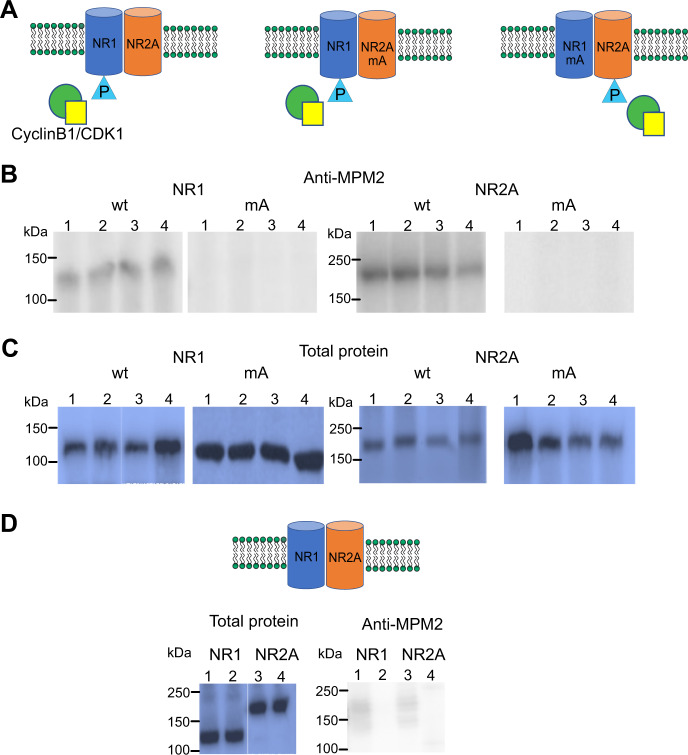
In vitro phosphorylation of NMDA receptors using a purified recombinant Cyclin B1/CDK1 complex. **a** Cartoon illustrating the NMDA subunit combinations purified from cells in interphase. The cylinders represent the subunits of the NMDAR. The triangles simulate the phosphorus given by the cyclin B1/CDK1 complex (green circle/yellow square). The subunits with the alanine mutant (mA) are not phosphorylated by the complex. **c**, **d** Purified NR1wt/NR2Awt, NR1mA/NR2Awt and NR1wt/NR2AmA were incubated with a purified recombinant CyclinB1/CDK1 complex (see “Materials and methods” section) for an in vitro phosphorylation assay. **b** Blots using the MPM2 antibody. NR1wt blot shows in lanes 1–2 replicas of NR1wt from the combination NR1wt/NR2Awt; and lanes 3–4 replicas of NR1wt from combination NR1wt/NR2mA. NR1mA blot shows in lanes 1–4 replicas from the NR2A purified from the combination NR1mA/NR2Awt. NR2Awt blot shows in lanes 1–2 replicas of NR1wt from the combination NR1wt/NR2Awt; and lanes 3–4 replicas from the NR2A purified from the combination NR1mA/NR2Awt. Finally, NR2AmA blot shows in lanes 1–4 replicas from the NR2A purified from the combination NR1wt/NR2AmA. **c** Blots show in the same order the total protein content identified by Coomassie staining. **d** Blots show the total protein (left) and western blot using the MPM2 antibody (right) from the combination NR1wt/NR2Awt which was not incubated with the phosphorylation cocktail containing the recombinant purified Cyclin B1/CDK1 complex. Lanes 1–2 replicas from NR1wt and lanes 3–4 replicas from NR2Awt. This was used to assess the background phosphorylation of NMDAR purified from cells in interphase.

Purified combinations of NR1wt/NR2Awt, NR1mA/NR2Awt, and NR1wt/NR2AmA were incubated with the Cyclin B1/CDK1 phosphorylation cocktail for 15 min and later immunoprecipitated to explore which NMDAR subunits were recognized by the MPM2 antibody. As illustrated in Fig. 3a, only wild type NR1 and NR2A were phosphorylated by the recombinant Cyclin B1/CDK1 purified complex as reported by the MPM2 antibody. As a control to assess background phosphorylation, the combination NR1wt/NR2Awt was purified but not incubated with the Cyclin B1/CDK1 phosphorylation cocktail (Fig. 3d). As previously shown (Fig. 2c), NMDAR purified from cells in interphase were not recognized by the MPM2 antibody (Fig. 3b, c and Supplementary Fig. 3 shows full-length blots and densitometry).

These results strongly suggest that the cyclin B1/CDK1 complex is responsible for the phosphorylation of NR1 and NR2A during mitosis. Furthermore, the fact that the NR1mA and NR2AmA are not in vitro phosphorylated by the recombinant cyclin B1/CDK1 complex shows that the phosphorylation is taking place in the putative phosphorylation site identified in silico and that serines S584 in NR1 and S580 in NR2A are the target of the complex. Even more, the negligible amounts of NMDAR phosphorylated in asynchronous cells (about 2%, Supplementary Fig. 3C) clearly shows that the receptors are phosphorylated in mitosis.

### Phosphorylation of NMDAR by cyclin B/CDK1 induces transient whole-cell currents and calcium influx

**Fig. 4:**
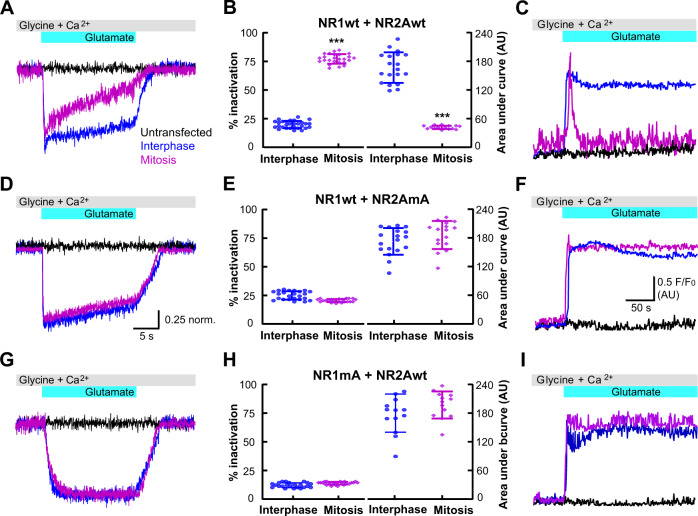
The single alanine mutation of NMDA receptors prevented transient calcium entry during mitosis. **a**, **d**, **g** Representative current traces obtained with glutamate applications at a membrane potential of −60 mV. Traces were obtained in transfected HEK293 cells in interphase (blue) or in mitosis (pink); non-transfected cells (black lines) were used as a negative control in mitosis (**a**) and in interphase (**d**, **g**). **c**, **f**, **i** Representative traces of calcium measurements. Traces were obtained in HEK293 cells transfected in interphase (blue) or in mitosis (pink); black traces were obtained in non-transfected cells in interphase (**c**) and in mitosis (**f**, **i**). The upper bars (gray and cyan) indicate the application of 100 µM glutamate, 10 µM glycine, and 2 mM calcium. **b**, **e**, **h** Percentage of inactivation (left) and quantification of area under the curve (right) calculated for HEK293 cells transfected with the indicated combinations of NMDAR-subunits at the top. In the graphs each blue circle represents an interphase cell and each pink rhombus a mitotic cell from seven independent experiments (at least ten cells measured in each independent experiment). The percent of inactivation for each current trace was obtained from the difference between the normalized current at the peak minus the remaining current measured at the second before glutamate was removed. The area under the curve was calculated by integrating under each of the calcium measurement traces. Data represent mean ± standard deviation with significance set at ***p < 0.0001, analyzed by two-tailed Student’s *t*-test in **b**, **e**, and **h**.

We characterized first the perforated-patch whole-cell currents and the calcium influx in HEK293 cells transfected with wild-type NR1wt + NR2Awt (Fig. 4a–c). As anticipated from our calcium measurements (Fig. 1d, e), inward currents evoked by glutamate were transient during mitosis, i.e., NMDA current inactivation was enhanced during mitosis with wild-type receptors (Fig. 4a, b).

To evaluate the role of serine 584 (NR1) and 580 (NR2A) in enhanced inactivation during mitosis, we explored the modulation of the alanine mutants expressed in HEK293 cells. A single mutation on either NR1 or NR2A was enough to prevent the enhanced inactivation during mitosis (Fig. 4d–i). Mixing wild-type NR1 (NR1wt) with alanine mutant-NR2A (NR2AmA) or NR1mA with NR2Awt produced identical results (Fig. 4d–i). Evidently preventing the phosphorylation of one of the NMDAR-subunits (NR1 or NR2A) suffices to eliminate the effect of cyclin B/CDK1 phosphorylation on NMDAR function during mitosis.

**Fig. 5:**
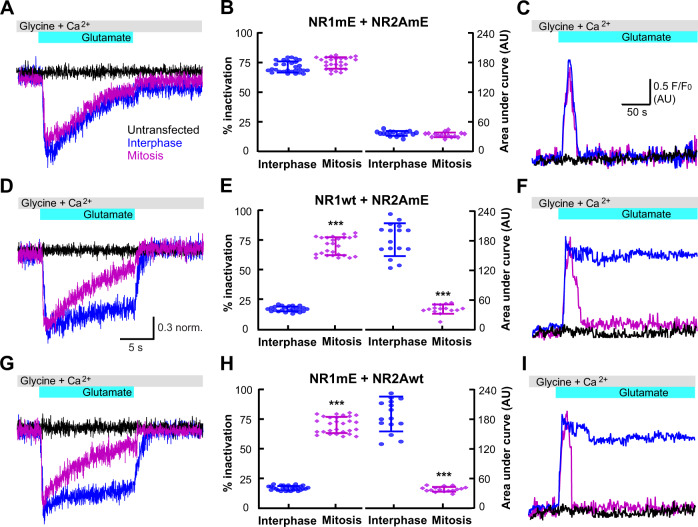
Interphase calcium entry in the phosphomimetic mutants emulated the transient pattern of the wild-type NMDAR-subunits in mitosis. **a**, **d**, **g** Representative currents recorded at −60 mV from transfected-HEK293 cells in interphase (blue traces) or in mitosis (pink traces); the black traces was obtained in nontransfected cells in mitosis (**a**) and in interphase (**d**, **g**). **c**, **f**, **i** Representative traces of calcium measurements in HEK293 cells transfected in interphase (blue) and in mitosis (pink); black traces were obtained in nontransfected cells in interphase (**c**) and in mitosis (**f**, **i**). The upper bars (gray and cyan) indicate the application of 100 µM glutamate, 10 µM glycine and 2 mM calcium. **b**, **e**, **h** Percentage of inactivation (left) obtained from traces of currents and the area under the curve (right) obtained from traces of calcium measurements. The combinations of transfected NMDAR-subunits are indicated above the graphs. In the graphs each blue circle and each pink diamond correspond to one cell from ten independent experiments. All data obtained from at least three independent transfections for each condition and measurements were obtained from at least 20 cells from each independent transfection. Data represent mean ± standard deviation with significance set at ***p < 0.0001, analyzed by two-tailed Student’s *t*-test in **b**, **e**, and **h**.

To further characterize the effects of cyclin B/CDK1 phosphorylation on NMDAR activity, we produced two phosphomimetic mutants (mE) in which serines 584 (NR1) and 580 (NR2A) were replaced by glutamic acid which would mimic phosphorylated serine. Expressing the double phosphomimetic mutants NR1mE + NR2AmE resulted in enhanced NMDA current inactivation during both interphase and mitosis (Fig. 5a–c). Surprisingly, phosphorylation of both subunits were required simultaneously to preserve the enhanced inactivation during mitosis, since the combination of NR1wt + NR2AmE (Fig. 5d–f) or NR1mE + NR2Awt (Fig. 5g–i) did not showed enhanced inactivation and transient calcium influx in interphase, only during mitosis when endogenous CDK1 would be expected to phosphorylate the alternate subunit. These results were consistent with our previous experiments showing that the presence of a single alanine mutant (either on NR1 or NR2A) was enough to prevent changes of NMDAR kinetics observed during mitosis. Notably, the double alanine mutant does not reach the plasma membrane, indicating that the double subunit mutation interferes with receptor trafficking to the plasma membrane. Total internal reflection fluorescence microscopy (TIRFM) experiments clearly showed that the double mutant NR1mA + NR2AmA did not reach the plasma membrane (Supplementary Fig. 4A). This observation was confirmed by fluorescence microscopy analysis of the plasma membrane (Supplementary Fig. 4B) and biotinylation assays (Supplementary Fig. 4C). For this reason, we did not investigate further the combined double alanine mutant.

### Phosphorylation by CDK1 modulates activity-dependent run-down of NMDAR

The most striking difference between phosphorylated and dephosphorylated NMDAR was the activity-dependent inactivation, thus we explored why phosphorylation enhanced channel inactivation.

**Fig. 6:**
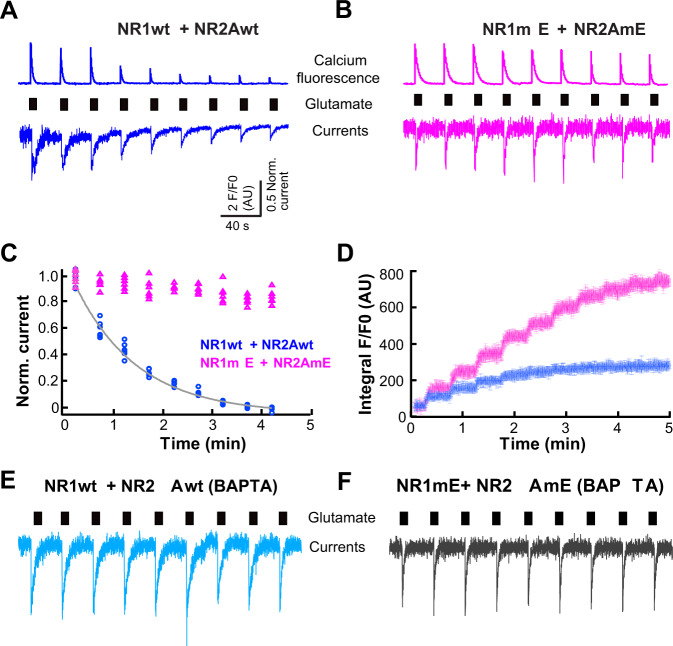
The NR1mE + NR2AmE phosphomimetic mutants have altered activity-dependent run-down. **a** Perforated patch clamp currents (below) and calcium measurements (above) obtained with repeated brief applications of 100 μM glutamate applied for 10 s every 500 s in HEK293 cells expressing wild-type NR1wt + NR2Awt NMDAR-subunits during interphase. **b** Similar experiments in cells expressing the phosphomimetic mutants. **c** Normalized whole-cell currents obtained from cells expressing the wild-type NMDAR-subunits (blue circles) or phosphomimetic mutants (pink triangles). The solid line is a fitted single-exponential decay (time constant *τ* = 1.3 min). **d** Mean with standard deviation of fluorescence (*F*/*F*0) integral from five independent measurements on cells expressing wild-type NMDAR-subunits (blue) or phosphomimetic mutants (pink). **e** Whole-cell experiments similar to those in **a** carried out with 10 mM BAPTA in the pipette for the wild-type NMDAR and **f** for the NR1mE + NR2AmE phosphomimetic mutants. All data obtained from at least three independent transfections for each condition and measurements were obtained from at least 20 cells from each independent transfection.

Whole-cell perforated-patch currents recorded with wild-type NR1 + NR2A subunits showed an activity-dependent run-down over repeated applications of glutamate (Fig. 6a). Simultaneous measurements of current and calcium (with FuraRed) showed that run-down of current paralleled the reduction of calcium influx (Fig. 6a). The activity-dependent run-down was not observed in cells expressing the phosphomimetic mutants NR1mE + NR2AmE (Fig. 6b). Thus, even though the NR1mE + NR2AmE shows faster inactivation during a single glutamate stimulus (Fig. 5) compared to wild-type NMDAR (Fig. 4), the repeated stimulation resulted in less activity-dependent run-down in the phosphomimetic mutants. This is more evident when comparing the relative amplitude of current triggered by repeated applications of glutamate (Fig. 6c). Since the phosphomimetic mutants inactivates faster during glutamate stimulation, one possible mechanism for reduced activity-dependent run-down is the reduction in calcium influx.

The inactivation of the NMDAR is a well characterized phenomenon in which calcium and calmodulin play critical roles^42^. To explore the role of calcium influx in NMDAR inactivation, we conducted experiments with cells expressing the wild-type receptors, loaded with the calcium chelator BAPTA that minimized any effects of calcium influx in NMDAR inactivation. With intracellular BAPTA, wild-type NMDAR lost their activity-dependent run-down, indicating that calcium influx was responsible for the run down (Fig. 6e). As expected, the activity of the phosphomimetic mutants was not altered using BAPTA (Fig. 6f). Activity-dependent run-down of NMDAR is a phenomenon that has been previously reported for NMDAR of dopaminergic neurons^43^.

Activity-dependent run-down plays a crucial role in determining the amount of calcium entering the cell over time during sustained or repetitive agonist stimulation. The integral of calcium fluorescence increases faster and is higher over time in the phosphomimetic mutants compared to wild-type NMDAR (Fig. 6d) suggesting that more calcium enters the cell overtime.

### The expression of NMDAR phosphomimetic mutations results in altered mitotic index, fragmentation of pericentriolar material and lagging chromosomes

**Fig. 7:**
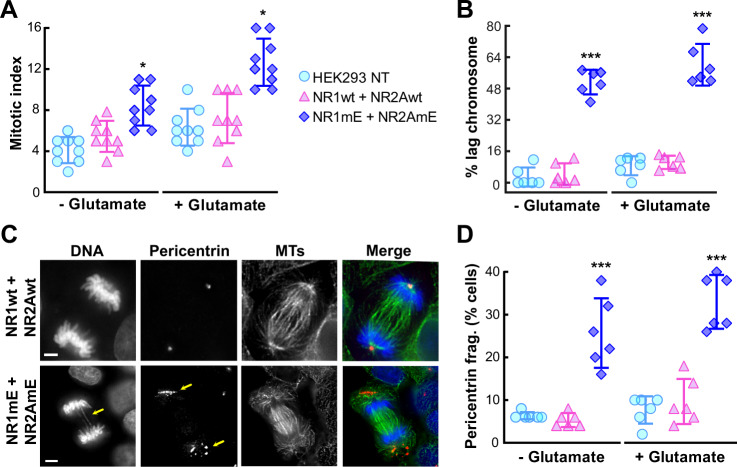
Cells transfected with phosphomimetic mutant NMDA receptors show an increase in mitotic index, alterations in chromosome segregation and centrosomes fragmentation defects. **a** Mitotic index quantification of HEK293 cells transfected with NMDAR or nontransfected (NT). Cells were divided into two groups and incubated with glutamate (+Glutamate) or without glutamate (−Glutamate). *n* = 900 cells per condition of three independent experiments, each symbol represents the percentage of cells in mitosis for a total count of 100 cells. **b** Percentage of anaphase cells with lagging chromosomes in HEK293 cells nontransfected (NT) and transfected with mutants of NMDAR-subunits. Cells were divided into two groups, those that were incubated with glutamate (+Glutamate) and those that were not incubated with glutamate (−Glutamate). *n* = 300 cells per condition of three independent experiments, each symbol represents 50 cells. **c** Mitotic spindles of HEK293 cells during anaphase: cell transfected with wild-type subunits (top) or phosphomimetic subunits (bottom). Cells were fixed and immunostained with antibodies against pericentrin and tubulin (MTs), DNA was visualized with DAPI. The yellow arrow in the DNA frame indicates the presence of chromosome lagging in the phosphomimetic mutants; the yellow arrows in the pericentrin frame indicate fragmentation of pericentriolar material. Scale bars: 5 μm. **d** Percentage of anaphase cells with pericentrin fragmentation in HEK293 cells nontransfected (NT) and transfected with mutants of NMDAR-subunits. Cells were divided into two groups, those that were incubated with glutamate (+Glutamate) and those that were not incubated with glutamate (−Glutamate). *n* = 300 cells per condition of three independent experiments, each symbol represents 50 cells. Data were analyzed by one-way ANOVA followed by Bonferroni multiple comparisons tests; data are presented as mean ± standard deviation with set at ***p < 0.0001, **p < 0.01, or *p < 0.05.

The selective phosphorylation of both subunits of the NMDAR by cyclin B/CDK1 during mitosis and the lack of phosphorylation during interphase suggested, that the receptor is dephosphorylated after leaving mitosis and phosphorylated again prior to entering mitosis at some stage during the next cell cycle. Such strong modulation of NMDAR functionality prompted the idea that perturbing this flip-flop regulation might affect cell cycle, particularly mitosis. To explore this hypothesis, we transfected HEK293 cells with the double phosphomimetic mutants to mimic constitutive phosphorylation of the NMDAR during the entire cell cycle. We observed several alterations in mitotic cells expressing the double phosphomimetic mutants. The phosphomimetic mutants increased the mitotic index (Fig. 7a), an effect that was exacerbated by maintaining cells in the presence of the NMDAR agonist glutamate (Fig. 7a). Changes of the mitotic index were not observed in nontransfected cells and cells expressing the wild-type subunits (Fig. 7a). The expression of NR1mE + NR2AmE phosphomimetic mutants also tripled the percentage of cells with lagging chromosomes during anaphase (Fig. 7b), promoted the appearance of multipericentrin foci (Fig. 7c), augmented fragmentation of pericentriolar material (Fig. 7d) led to defective mitotic spindles and the formation of micronuclei (Supplementary Video 1).

In short, in cells expressing the phosphomimetic mutants we have seen a persistent calcium entry with decrease activity-dependent run down of NMDAR (Fig. 6) and several abnormalities in mitosis (Fig. 7 and Supplementary Video 1). These abnormalities highlight the importance of the dynamic control of the phosphorylation state of NMDAR in the different phases of the cell cycle and the tight regulation of intracellular calcium levels during cell division.

### The mitotic checkpoint is not affected by overexpression of NMDAR phosphomimetic mutant

**Fig. 8:**
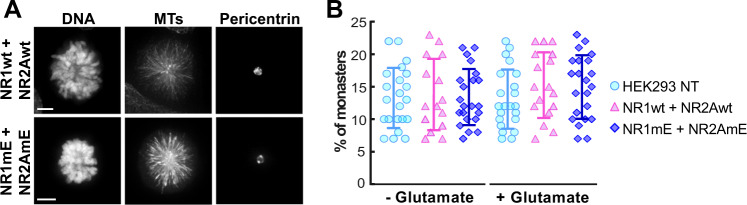
The percentage of monasters in monastrol-arrested cells is the same between cells transfected with wild-type NMDAR and phosphomimetic mutants (mE). **a** Representative images of cells arrested as monasters after 4 h treatment with monastrol. Scale bar: 5 µm. **b** Quantification of percentage of monasters with respect to the total number of cells. There is no significant difference between wild-type NMDAR-subunits (NR1wt and NR2Awt), phosphomimetic mutants (NR1mE and NR2AmE) and alanine mutants (NR1mA and NR2AmA) transfected cells. Each symbol represents a field of view of 100 cells from three independent experiments. Data were analyzed by one-way ANOVA followed by Bonferroni multiple comparisons tests; data are presented as mean ± standard deviation with no significance.

One of the main causes for the appearance of lagging chromosomes is a malfunction of the spindle assembly checkpoint (SAC; also known as mitotic checkpoint, MC). The SAC is a surveillance system comprised of several proteins that arrest cells before anaphase until all chromosomes have achieved proper attachments to microtubules. To probe the SAC status, we used the double phosphomimetic mutants in experiments using monastrol. This inhibitor is a cell-permeable molecule that arrests cells in prometaphase by specifically inhibiting Eg5^44^, a member of the kinesin-5 family. Eg5 can crosslink and slide antiparallel microtubules to separate centrosomes at the beginning of mitosis to form a bipolar spindle. Monastrol inhibits the ATPase activity of Eg5, blocking the separation of the centrosomes and creating a monopolar spindle, also known as monaster. These monasters have both centrosomes at the center of the cell with microtubules (MTs) emanating radially from them, and with the DNA around the pair of centrosomes (Fig. 8a). After 4–5 h in the presence of monastrol, cultured cells show a substantial number of monasters. During this treatment, two events can occur: (1) Cells exit mitosis without going through metaphase–telophase, or (2) Cells are arrested for several hours in prometaphase. If cells exit mitosis, it suggests that the mitotic checkpoint is not working properly. Therefore, the percentage of “monaster cells” in a monastrol-treated culture, is an index of how many cells have their mitotic checkpoint working correctly; thus, a higher percentage of “monaster cells” indicates that the mitotic checkpoint is not affected.

During monastrol treatment no significant differences of “monaster cells” were observed when expressing wild-type NMDAR or the phosphomimetic mutants (Fig. 8a, b). This result indicates that the expression of the NR1mE + NR2AmE phosphomimetic mutants do not alter the mitotic checkpoint but must be affecting phases prior to mitosis.

Based on the evidence gathered in the present study, we propose a cycling process of phosphorylation (by the cyclin B/CDK1 complex), in which during mitosis NMDAR are phosphorylated, and during interphase NMDAR are dephosphorylated (by a yet unidentified phosphatase, most likely PP1 and PP2A). This process is essential to sustain a functional cell cycle in cells expressing the NMDA receptors. The phosphorylation/dephosphorylation cycling process of the NMDAR alters channel function and determines the amount of calcium influx into the cell. Phosphorylated NMDAR sustain higher calcium influx, which is might be deleterious if occurs during interphase but may be required during mitosis.

## Discussion

Cell proliferation is essential for tissue repair, growth, development, and reproduction. Alterations in the molecular mechanisms that control cell proliferation might lead to several disorders and illnesses. For example, enhanced cell proliferation is a characteristic of cancer cells. There are several studies that have correlated an increased function of NMDAR with enhanced proliferation of retinal epithelial cells and Jurkat cells^45,46^. Interestingly, the NMDAR natural agonist, glutamate, is readily available to many tissues, since micromolar concentrations are present in the bloodstream^47^. Thus, many cells outside the CNS that express NMDAR under pathological conditions such as cancer, may have active receptors stimulated by circulating glutamate.

However, none of these studies have delved into the function of these receptors during mitosis, the process that enables cell proliferation. Thus, our goal was to uncover how NMDAR regulate mitosis at single cell level. In the present study, we explored calcium entry via endogenous NMDAR in individual rat cortical astrocytes and we found that the kinetics of calcium entry were different in mitotic astrocytes compared to astrocytes in other phases of the cell cycle (Fig. 1a–c). We were able to reconstitute this phenomenon in HEK293 cells, a cell line that lacks endogenous NMDAR, by expressing NR1 + NR2A subunits (Fig. 1d–f). Our data suggested that NMDAR activity is differentially regulated in interphase and mitosis.

The main event triggering mitosis is the activation of cyclin B/CDK1. Therefore, we explored whether CDK1 phosphorylated NMDAR during mitosis. Through bioinformatics we identified serines 584 (NR1) and 580 (NR2A) as the residues phosphorylated by CDK1. Replacing these serines by alanines abrogated MPM2 antibody labeling (Fig. 2c, d), strongly suggesting those as the targeted residues. Because MPM2 antibody recognizes a phosphorylation consensus sequence used by all CDK, we conducted in vitro phosphorylation experiments using a recombinant purified CyclinB1/CDK1 complex and purified NMDAR. Our results indicate that NR1 and NR2A subunits are phosphorylated by the CyclinB1/CDK1 complex at serines 584 (NR1) and 580 (NR2A) (Fig. 3, for quantification of four replicas please refer to Supplementary Fig. 2).

Replacing any of these serines by alanine was enough to prevent the phenotype observed in mitosis (Fig. 4). In contrast, both NR1 and NR2A subunits must be phosphorylated in order to observe the transient calcium influx and enhanced inactivation found in mitotic cells (Fig. 5).

Interestingly, the double alanine mutant (NR1mA + NR2AmA) failed to reach the plasma membrane and was retained in intracellular compartments (Supplementary Fig. 2), suggesting a potential alteration in receptor trafficking. This phenotype was not explored further in the present study. Our results revealed a tight modulation of NMDAR subunits during the cell cycle mediated by phosphorylation carried by the cyclin B/CDK1 complex in mitosis and dephosphorylation by a yet unidentified phosphatase after leaving mitosis. This modulation is consistent with the phosphorylation/dephosphorylation regulation of many proteins involved in the modulation of mitosis^48^, where several kinases and phosphatases work together to regulate the entry, progression, and exit of mitosis. PP1 and PP2A are the main phosphatases active during mitosis and are essential for mitotic exit^49^. Since NMDAR are dephosphorylated after mitosis and PP1 and PP2A regulate the exit of mitosis, it is possible that these proteins also modulate dephosphorylation of NMDAR. Further research should be done on this topic to fully understand the phosphorylation/dephosphorylation cycling of NMDAR during cell cycle.

NMDAR phosphorylation changes the activity of the receptors, therefore modulating the intracellular calcium concentrations. Other groups have reported that changes in the activity or expression of potassium channels modifies glial cell proliferation and differentiation^50,51^. In fact, spinal cord astrocytes exhibit oscillations of K_V_ and K_IR_ channels throughout the cell cycle, which is associated to changes in the proliferation of these cells^52^. Furthermore, tumor necrosis factor-α (TNF-α), a known mitogen for astrocytes, causes membrane depolarization and reduced K_IR_ currents, suggesting that changes in membrane potential and ion currents are early markers for cell division onset^53^. Besides the role in proliferation, NMDAR have also been implicated in cell differentiation. In fact, an increase in NMDAR activation induces maturation of neuronal progenitor cells^10^. Therefore, the regulation of calcium entry through these receptors is important not only to glial cell proliferation but also for neuronal differentiation. Thus, our results open a new line of research where the phosphorylation-dependent changes in NMDAR activity throughout the cell cycle could influence differentiation/proliferation of glial cells.

Some reports have linked NMDAR to cancer, including breast cancer, small-cell lung cancer, ovarian cancer, neuroblastoma, and glioblastoma^12,54,55^. Such studies were performed in cell populations to check how the receptors affect proliferation and were not focused on mitotic events from a single-cell perspective. Here, we showed that the expression of NMDAR phosphomimetic mutants in single cells induced several modifications during mitosis, including increased mitotic index, augmented number of cells with lagging chromosomes during anaphase, fragmentation of pericentriolar material led to defective mitotic spindles and the formation of micronuclei (Fig. 7 and Supplementary Video 1). Noteworthy, the mitotic checkpoint was not affected by the expression of phosphomimetic mutants (Fig. 8a, b). These results support the hypothesis that the defects in mitosis described in the present study are produced at an earlier phase of the cell cycle, such as S or G2 but manifest during mitosis. This is consistent with the fact that NMDAR are not phosphorylated in interphase, only during mitosis (Fig. 2c). Forcing the presence of phosphorylated NMDAR in interphase (expressing the phosphomimetic mutants) results in the mitotic alterations listed above.

The presence of multipericentrin foci in cells expressing the phosphomimetic mutants, provided a hint of two likely molecular mechanisms; one that would take place in S phase with centrin, and another that would occur in G2 with pericentrin. The centrosomes are organelles containing two perpendicular centrioles and a surrounding matrix of proteins known as pericentriolar material (PCM). Centrosomes are duplicated during S phase in order to prepare the cell for the formation of a bipolar spindle during mitosis^56^. During G2, the PCM acquires new layers of proteins in a process known as centrosome maturation^57^, that is essential for proper formation of a bipolar spindle. Centrin, a calcium binding protein with 4 EF-hand domains, localizes to the centrosome and it is used as a marker for centriole dynamics in living cells^58^. Knockdown of centrin triggers centriole loss^59^. Calcium is important for the assembly of centrin^60^, and changes in intracellular levels of calcium correlate with an increased number and size of pericentrosomal spots^61^. On the other hand, pericentrin is a large coiled-coil protein that localizes to centrosomes and PCM, it is essential for centrosome maturation and mitotic spindle formation^62^. Pericentrin is also a calmodulin-binding protein^63^, and in *Drosophila* the interaction with calmodulin (CaM) is required for PCM organization. Disrupting the pericentrin–CaM interaction leads to PCM disorganization in neuronal cells^64^. Since centrin and pericentrin functions are affected by calcium and calmodulin, one feasible explanation is that the constitutive expression of the double phosphomimetic mutants show reduced activity-dependent run-down, which results in increased calcium influx (Fig. 6) in a phase prior to mitosis (S or G2) triggering defects in PCM formation that lead to the chromosome segregation defects observed in mitotic cells (Fig. 7b–d and Supplementary Video 1).

The most striking defect is the missegregation of parts of chromosomes or even whole chromosomes during anaphase. The increased rate of chromosome missegregation is known as chromosome instability (CIN), and it is the cause of aneuploidy^65^. CIN could induce tumorigenesis. Therefore, it is possible that these receptors participate in the origin and evolution of some cancers affecting chromosome segregation during mitosis due to altered phosphorylation by the cyclin B/CDK1 complex. Even more, CIN is correlated with drug resistance, metastasis, and poor prognosis in cancer patients^66^. Therefore, keeping the NMDAR phosphorylation state oscillating during each cell cycle, could be relevant for improving the poor prognosis in different types of cancer; including glioblastoma, the most common malignant primary brain tumor which is characterized by enclosing undifferentiated astrocytes^11,12^.

## Methods

### Plasmids

Cav1-mCherry was acquired from Addgene (#27705). NMDA receptors plasmids were acquired from Addgene: pCI-EGFP-NR1 wt (#45446) and pCI-SEP_NR2A (#23997).

### Site-directed mutagenesis

To introduce the alanine and glutamic acid mutations (S580mA/mE and S584mA/mE), site-directed mutagenesis was performed with the QuikChange^®^ II site-directed mutagenesis kit (Agilent Technologies); according to the manufacturer’s instructions using the following primers:

1. The prevent phosphorylation (alanine mutation) in pCI-EGFP-NR1wt:
  forward 5′-gtacctgctggaccgcttcgctccctttggccgattcaag-3′ and
  reverse 5′-cttgaatcggccaaagggagcgaagcggtccagcaggtac-3′.
2. To emulate phosphorylation (glutamic acid mutation or phosphomimetic mutation) in pCI-EGFP-NR1wt:
  forward 5′-gtacctgctggaccgcttcgagccctttggccgattcaag-3′ and
  reverse 5′-cttgaatcggccaaagggctcgaagcggtccagcaggtac-3′.
3. The prevent phosphorylation (alanine mutation) in pCI-SEP_NR2A:
  forward 5′-cttcgtttttgaatacttcgctcctgttggatacaacag-3′ and
  reverse 5′-ctgttgtatccaacaggagcgaagtattcaaaaacgaag-3′.
4. To emulate phosphorylation (glutamic acid mutation or phosphomimetic mutation) in pCI-SEP_NR2A:

forward 5′-cttcgtttttgaatacttcgagcctgttggatacaacag-3′ and

reverse 5′-ctgttgtatccaacaggctcgaagtattcaaaaacgaag-3′.

All constructs were fully sequenced before transfection in the Molecular Biology Unit at the Instituto de Fisiología Celular/UNAM. Underlined are the mutated codons.

### Cell culture of rat astrocytes

Primary cultures of cortical astrocytes from 7-day-old male Wistar rats were performed according to the protocol reported by McCarthy and Vellis^67^; cells were cultured in basal medium and cultures were used for experiments at 6–8 days after removing the animal. Animals were sacrificed following a strict protocol approved by our ethics and animal welfare commissions.

### Cell culture and expression of NMDA receptors

Human embryonic kidney 293 cells (HEK293; ATCC) were cultured using Dulbecco’s modified Eagle’s medium (DMEM) (GIBCO) supplemented with 10% (V/V) fetal bovine serum, 50 µg ml^−1^ penicillin–streptomycin and maintained at 37 °C in a humidified atmosphere with 5% CO_2_.

HEK293 cells expressing NR1 and NR2A subunits (1:1 ratio) were placed on coverslips coated with poly-lysine (Sigma). Transient transfection was performed using Lipofectamine 2000 (Invitrogen) according to manufacturer instruction using cells seeded to 80% confluence in Optimem medium (GIBCO). Cells were studied between 24 and 48 h post-transfection.

### Cell arrest

To increase the mitotic index for western blot experiments, HEK293 cells were arrested in mitosis using a double treatment with thymidine and nocodazole. Cells were: first, incubated with 2 mM thymidine for 14 h to arrest in G1-S phases, then washed with PBS and incubated for 4 h with supplemented DMEM medium (G1-S release time). Finally, cells were incubated with nocodazole at a concentration of 0.4 μg/ml.

For calcium measurements and electrophysiology experiments, 24–28 h post-transfected cells were arrested for 4–6 h with nocodazole at 0.4 μg/ml. All the electrophysiology and calcium measurements were performed in arrest-cultures. All solutions with calcium indicators or external solution contained nocodazole to maintain mitotic arrest. Cells in mitosis were identified by their condensed DNA or by their chromosomes aligned at the equator, by spherical cell morphology and by rupture of the nuclear membrane. To select interphase cells, cells were chosen flat, isolated, with noncondensed DNA and an intact nuclear membrane.

### Identification of NMDAR in western blots

Total protein from transfected HEK293 cells was extracted using RIPA buffer (50 mM Tris HCl, 150 mM NaCl, 1 mM EDTA, 1 mM phenylmethylsulfonyl fluoride, 1% v/v NP-40 and 0.25% w/v sodium deoxycholate, pH 7.5) supplemented with 1× Complete Mini Protease Inhibitor (Roche Applied Sciences) and 1× Halt Phosphatase Inhibitor Cocktail (Thermo Scientific).

Proteins from HEK293 cells transfected with NMDAR constructs were separated using SDS-Page with 10% acrylamide and transferred to the membrane using 110 V for 60 min in a wet chamber. Primary antibody was incubated with agitation at 4 °C overnight. The secondary antibody was incubated for two h with agitation at room temperature. Signal was acquired using X-ray films and digitalized and analyzed using ImageJ software. Full-length blots are included in Supp. Fig. 1.

For immunoprecipitations, lysates containing equal protein amounts were precleared for 1 h with Protein A/G beads (Thermo Scientific), incubated with primary antibodies overnight (anti-GFP, 1:1000 dilution) and then incubated with Protein A/G beads for an additional 4 h, all at 4 °C. Then, beads were rinsed three times with RIPA buffer and proteins were eluted with Laemmli sample buffer containing 5% β‑mercaptoethanol, boiled at 95 °C for 5 min. The proteins were analyzed using western blot: the molecular weight of GFP-NR1 is 132 kDa and of GFP-NR2A, 192 kDa. Stripping was carried out using Restore reagent (Thermo Scientific).

### Selective labeling of proteins located at the cell surface

Five 100 mm Petri dishes with HEK293 cells expressing the different NMDAR constructs were washed three times with ice-cold PBS, pH 8.0, to remove any contaminating proteins.

Cells were suspended at a concentration of ~10 million cells/ml in PBS, pH 8.0. 1 mg of sulfo-NHS-LC-Biotin (Thermo Scientific) per ml of reaction volume was added to the cells and incubated at 4 °C for 30 min. After this, cells were washed three times with ice-cold PBS and 100 mM glycine to quench any remaining biotinylation reagent. The cell surface proteins are now biotinylated on exposed lysine residues.

Biotinylated proteins were purified using the Thermo Scientific Pierce Cell Surface Protein Isolation Kit^®^ following manufactured instructions. For loading control of western blots, an aliquot of total protein (before purification of biotinylated protein) was immunoprecipitated using anti-GFP selective polyclonal antibody (Thermo Scientific, A-11122), and the immunoprecipitation kit (Abcam ab206996) following the manufacturer instructions (both NMDAR subunits had GFP fused). For the identification of the NMDAR on the western blots, the selective rabbit monoclonal antibody (ab109182) was used for NR1 and antibody (ab133265) for NR2A (Abcam).

### In vitro phosphorylation of NMDAR

Cultured HEK293 cells expressing the following combinations: NR1wt/NR2Awt, NR1wt/NR2AmA, and NR1mA/NR2A-wt were scraped off the dish, washed, and resuspended in 5 ml of buffer containing 60 mM KCl, 2 mM MgCl_2_, 0.2 mM CaCl_2_, 20 mM imidazole, pH 7.0 and maintained iced cold. Cells were homogenized and centrifuged at low speed (10,000 × *g*, 30 min). The supernatants were filtered through glass wool, and centrifuged at high speed (50,000 × *g*, 120 min). The pellets were washed three times with 1 ml of the following buffer: 120 mM KCl, 0.2 mM CaCl_2_, 20 mM imidazole, pH 7.0 and centrifuged at 10,000 × *g* for 30 min. Pellets were resuspended and adjusted to final protein concentration of 2 mg/ml. Aliquots of 100 µl were incubated with the CyclinB1/CDK1 PRECISIOⓇ Kinase kit (Sigma-Aldrich, Saint Louis, MO) using the in vitro phosphorylation buffer provided by the manufacturer and following the manufacturer instructions. After the phosphorylation assay concluded (15 min), the NMDA receptors were immunoprecipitated following the protocol described in the section “Identification of NMDAR in western blots” section, using selective antibodies (ab109182) for NR1 and (ab133265) for NR2A (Abcam). To identify total protein SDS-PAGE gels were stained with Coomassie blue (Sigma-Aldrich). Gels were transferred to membranes to identify Cyclin B1/CDK1 in vitro phosphorylated NMDAR by western blot as described earlier using the MPM2 antibody. For control of the Cyclin B1/CDK1 in vitro phosphorylation, immunoprecipitated NMDAR not treated with the phosphorylation assay were analyzed to determine the levels of background phosphorylation by western blot using the MPM2 antibody as described above.

### Whole-cell currents in the perforated-patch mode

Coverslips were mounted on an open perfusion chamber (TIRF Labs, Cary, NC). A continuous bath perfusion system was maintained to dilute the Picospritzer III perfused solution in areas distant from the studied single cell. An EPC-10 (Heka Electroniks, Germany) patch-clamp amplifier was used for whole-cell recordings. The patch-clamp pipettes were prepared from Corning 7052 glass and had a resistance of 1–5 MΩ when filled with the pipette solution (see below). An Ag/AgCl electrode was connected to the bath solution via a KCl-agar bridge. Glutamate was applied using a rapid local perfusion system (Picospritzer III, Parker Hannifin, USA) directly on the cell studied. Amphotericin B was dissolved in 50 μl DMSO to give a 60 mg ml^−1^ stock solution and stored at −20 °C until use. The amphotericin B stock solution was dissolved at a final concentration of 0.24 mg ml^−1^ in a syringe containing the pipette solution: 125 mM CsMeSO_3_, 15 mM CsCl, 0.5 mM CaCl_2_, 5 mM BAPTA adjusted to pH 7.2 and 305 mOsm. The syringe was used to filled patch-clamp pipettes. The extracellular control solution consisted of 160 mM NaCl, 2.5 mM KCl, 2 mM CaCl_2_, 10 mM glucose, 10 µM glycine, 10 mM HEPES adjusted to pH 7.3 and 325 mOsm.

The voltage-clamp protocol maintains a holding potential of −60 mV while applying glutamate to activate NMDAR currents for the duration indicated in each figure. Nontransfected HEK293 cells were used as control.

For run-down experiments, HEK293 cells were transfected with wild-type NR1wt + NR2Awt or with the NR1mE + NR2AmE phosphomimetic mutants and were studied in the perforated-patch and whole-cell configuration of the patch-clamp. In experiments designed to evaluate the role of intracellular calcium, the patch-clamp pipette contained 10 mM BAPTA (in whole-cell configuration). For whole-cell experiments the pipette solution consisted of 150 mM KCl, 5 mM NaCl, 200 µM EGTA, and 10 mM HEPES adjusted to pH 7.2 and 325 mOsm. The extracellular solution was the same as that for perforated-patch studies.

All currents were imported into Igor pro v. 7 (Wavemetrics, Oregon) for further analysis and plotting. Final figures were created with Adobe Illustrator (Adobe Systems).

### Calcium measurements and confocal microscopy

Calcium imaging was performed on 25 mm coverslip with astrocytes non-transfected or HEK293 cells transfected with the plasmid of choice indicated in the figure legends. Astrocytes and HEK293 cells were loaded with Fura-Red^TM^AM (Molecular probes). Both calcium indicators were used at 2 μM final concentration in calcium-free Krebs solution and incubated for 40 min at 25 °C followed by 15 min in calcium-free Krebs solution without indicator. Calcium-free Krebs solution contain: 125 mM NaCl, 2.5 mM KCl, 1 mM NaH_2_PO_4_, 20 mM HEPES, 11 mM glucose, 500 μM EGTA, and 10 μM glycine adjusted to pH 7.4. To activate NMDA receptors 10 μM glycine + 100 μM of glutamate (Sigma-Aldrich) was used with 2 mM of calcium (CaCl_2_).

To determine the phase of the cell cycle, DNA of astrocytes and HEK293 cells was stained with Syto^TM^59 (Invitrogen). GFP fluorescence was used to localize GFP-NMDAR. Images were collected with an Olympus Fv10i confocal microscope equipped with a UPLSAPO 60 × 1.35 NA oil immersion objective using the following exciting wavelengths: GFP-tagged NMDAR-subunits, 489 nm; Syto^TM^59, 653 nm; Fura-Red^TM^AM, 472/433 nm for the unbound calcium channel and the bound calcium channel, respectively. The confocal acquisition window was set to 512 × 512 pixels which allowed to acquire one image per second for calcium indicators and only one image for DNA and GFP-NMDAR. Images were analyzed with the microscope software FV10ASW and Imaris 8.2 software (Bitplane). To calculate the *F*/*F*_0_ values, the fluorescence intensities of FuraRed bound to calcium were divided by the fluorescence intensities of FuraRed not bound to calcium for each time point; this was called *F*. Each of these *F* values was then divided by the highest value, called *F*_0_, in the time series.

To obtain the *F*/*F*_0_, the fluorescence of the calcium-bound indicator was divided by the fluorescence of the noncalcium-bound indicator in each second, and then plotted using the software GraphPad Prism (GraphPad Software Inc).

### Total internal reflection microscopy (TIRFM)

TIRFM used an Olympus IX81 inverted microscope equipped with an oil immersion 100× PlanApo objective with a numerical aperture of 1.45. The microscope was equipped with a LG-TIRFM (TIRF Labs, Cary, NC) system coupled to an Argon-Helium laser with three lines; 488, 543, and 633 nm as previously described^68^. The 488 nm line was used to excite GFP (NMDA receptors) and the 543 to excite Cav1-mCherry. We introduced an Optosplit image splitter (Cairn Research, Kent, UK) before the camera (Ixon Andor) to collect simultaneous images from GFP (525 nm) and mCherry (580 nm).

### Immunofluorescence cytochemistry

Cells were fixed in 4% paraformaldehyde in PBS at 37 °C for 15 min and permeabilized in 0.5% Triton X-100 for 5 min at room temperature. Tubulin and centrosomes were labeled using antitubulin monoclonal DM1-alpha (1:500, Sigma) or antipericentrin (1:500, Abcam) applied overnight at room temperature. Alexa-fluor 568 and 647 secondary antibodies against mouse and rabbit were used to detect the antitubulin and antipericentrin antibodies. To label the cell membrane, cells were incubated 30 min at room temperature with Phalloidin-Alexa-fluor-568 after the treatment with the secondary antibodies. After phalloidin treatment, cells were washed three times ten minutes each with 1× PBS at room temperature. Coverslips were mounted using ProLong® Diamond containing DAPI (Molecular Probes) and cured for 24 h. Fluorescence images were collected as 0.5-μm Z-stacks on a Deltavision microscope system (Applied Precision/GEHealthcare) using a 60 × 1.42 NA lens (Olympus). Images were deconvolved using SoftWorx 5.0 (Applied Precision/GEHealthcare), and representative images are presented as a flat Z-projection. For assessment of spindle profiles and mitotic index, cells were stained as above to visualize tubulin and pericentrin. Mitotic stages were scored by hand using a Nikon FX-A microscope with a 60 × 1.4 NA lens.

For monopolar spindle experiments, cells were treated with monastrol (AG Scientific) for 4 h prior to glutamate treatment. The mix glutamate and monastrol were left in the media for additional 30 min before fixation. Number of monasters was determined manually.

### Time lapse microscopy

For live-cell imaging, HEK293 cells cultured in glass-bottom 24 multiwell plates from Ibidi were transfected with phosphomimetic mutants of NMDAR and H2B-mCherry plasmids using Lipofectamine 2000 according to manufacturer’s instructions. Two transfections per NMDA receptors were performed. After 36 h, half of the wells were treated with glutamate/glycine for 15 min. Before imaging, cells were switched to 37 °C CO_2_ independent media (10% FBS). Live-cell images were acquired using a GE InCell 2500 system with a 20× lens. Time-lapse images were collected as 20 µm *Z*-stack series, with 4 µm *Z*-spacing, and at 5 min time intervals for 24 h. Images were deconvolved using the InCell software and processed with Fiji.

### Statistics and reproducibility

Data were analyzed by one-way ANOVA followed by Bonferroni multiple comparisons tests or by two-tailed Student’s *t*-test (GraphPad Prism). Unless otherwise indicated, data are presented as means ± standard deviation with significance set at ***p < 0.0001, **p < 0.01, or *p < 0.05. All statistical analysis was performed with Prism v6 (GraphPad, San Diego, CA). All experiments that were not subjected to statistical analysis were repeated at least three times with similar results.

## Supplementary information

Supplementary Information

Description of Additional Supplementary Files

Supplementary Video 1

Supplementary Data 1

## Data Availability

All full length unedited western blots are included in supplementary figures. The data from imaging calcium measurements, cell cycle and electrophysiology is included in Supplementary Data 1. Plasmids containing the cDNAs for the NMDAR subunits (wild type and all mutants produced for this study) are available upon request.
