# Supplementary material for: Phosphorylation of NMDA receptors by cyclin B/CDK1 modulates calcium dynamics and mitosis
Source: Commun Biol. 2020 Nov 12;3:665. doi: 10.1038/s42003-020-01393-3 (PMC7665045; doi:10.1038/s42003-020-01393-3)
Supplement: Supplementary file 1 — Supplementary Information [file 42003_2020_1393_MOESM1_ESM.pdf]

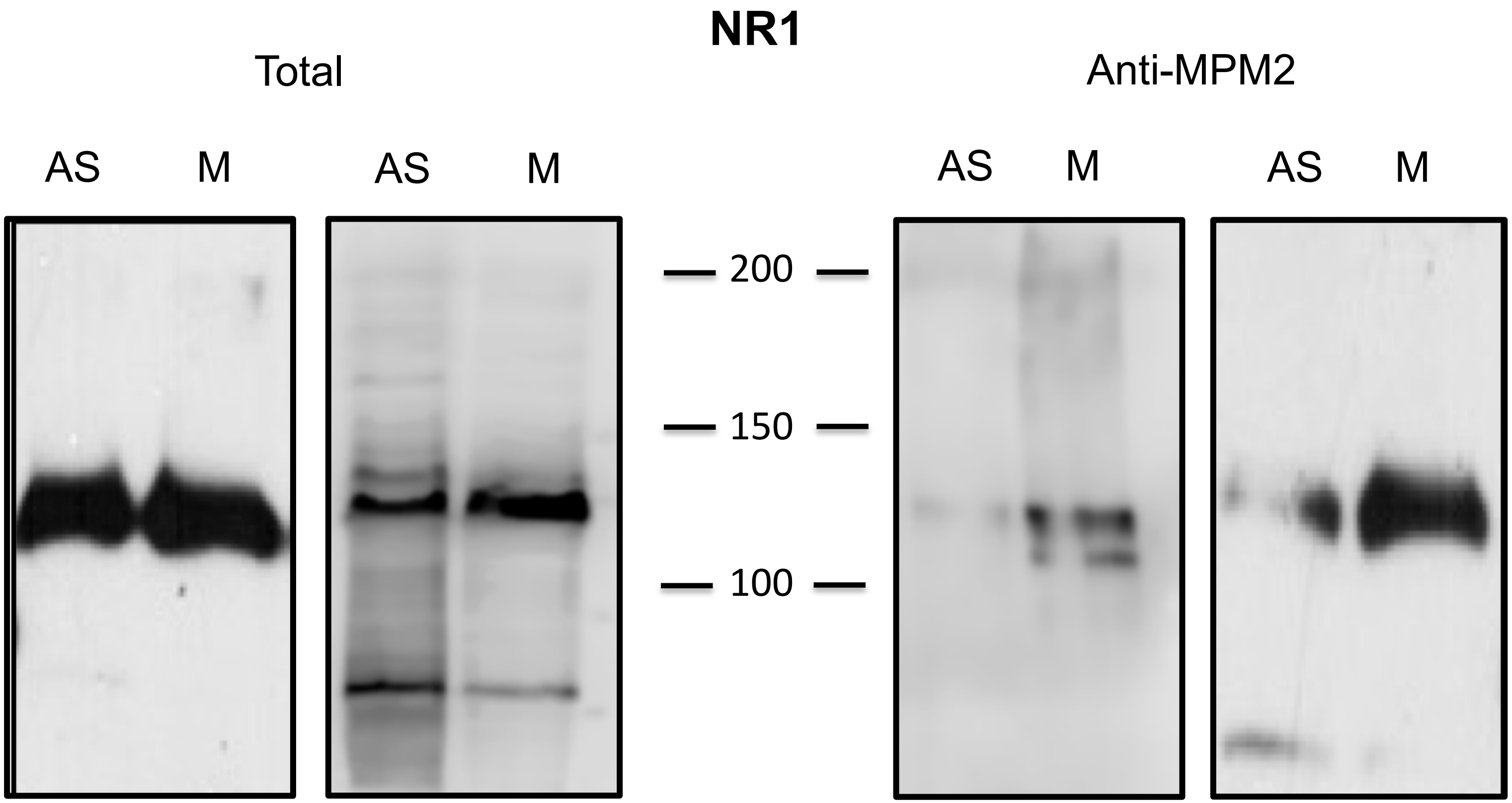

NR1
Total
Anti-MPM2
AS
M
AS
M
AS
M
AS
M
200
150
100

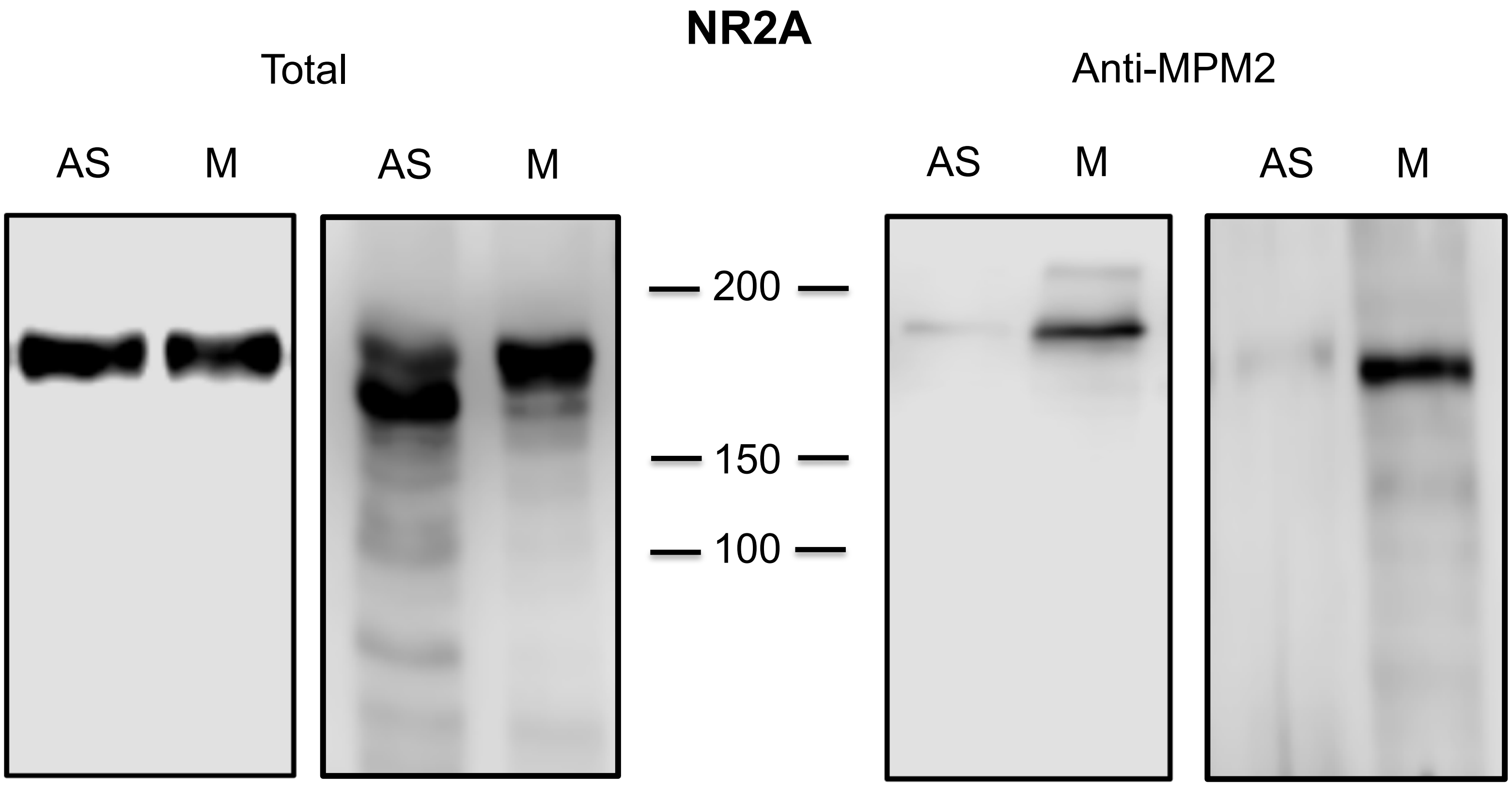
NR2A
Total
Anti-MPM2
AS
M
AS
M
200
150
100
AS
M
AS
M

Supplementary figure 1. Full-length western blots of wild type NMDA receptors in asynchronous cells (AS) and mitotic cells (M). The panels show full-length western blots with two replicas of each subunit of NMDAR (NR1 and NR2A). Left panel shows total protein (anti-GFP) and right panel shows the same blot with anti-MPM2 antibody. Blots correspond to WB cuts illustrated in Fig. 2C.

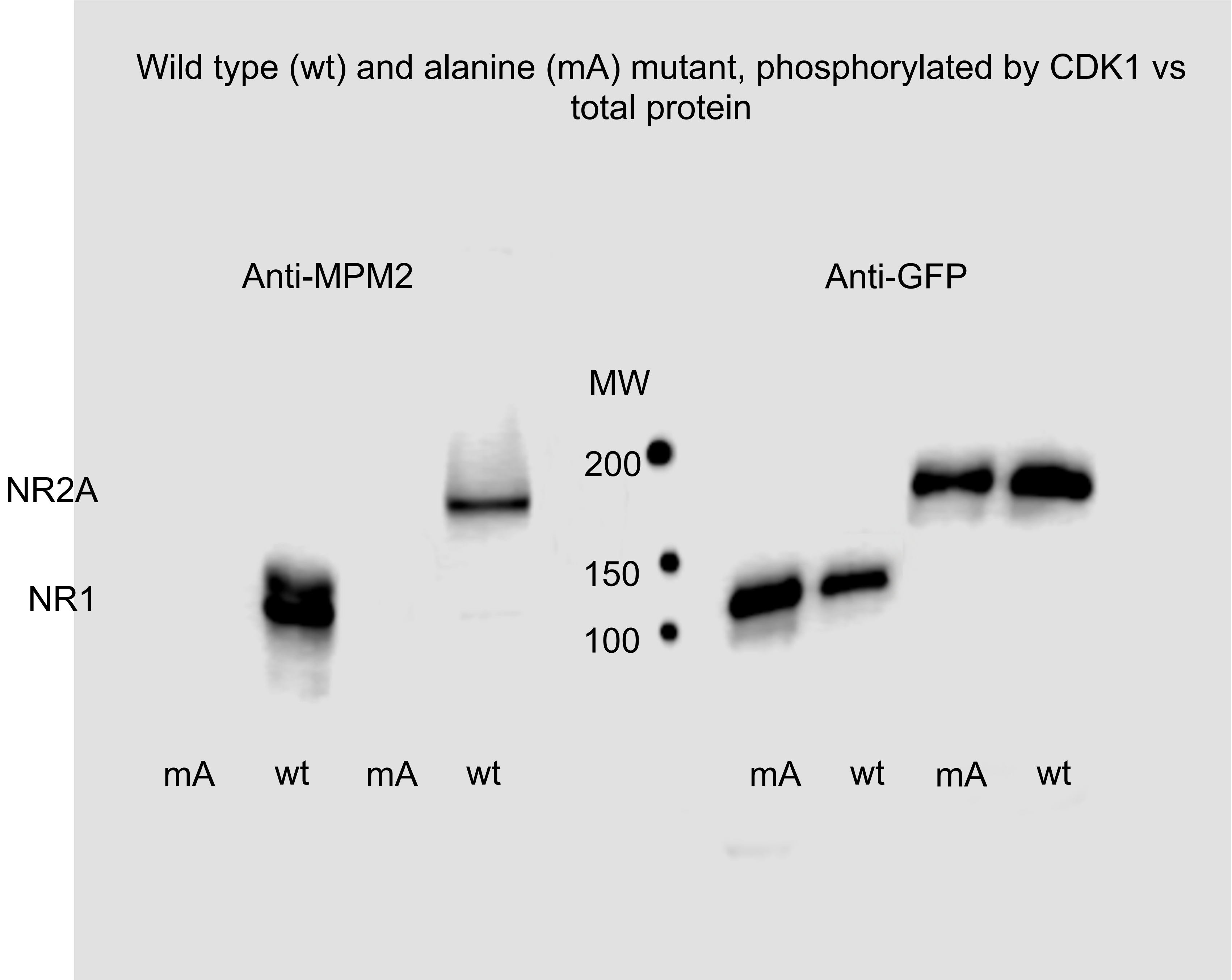
Wild type (wt) and alanine (mA) mutant, phosphorylated by CDK1 vs total protein
Anti-MPM2
Anti-GFP
MW
200
150
100
NR2A
NR1
mA
wt
mA
wt
mA
wt
mA
wt

Wild type (wt) and alanine (mA) mutant, phosphorylated by CDK1 vs total protein

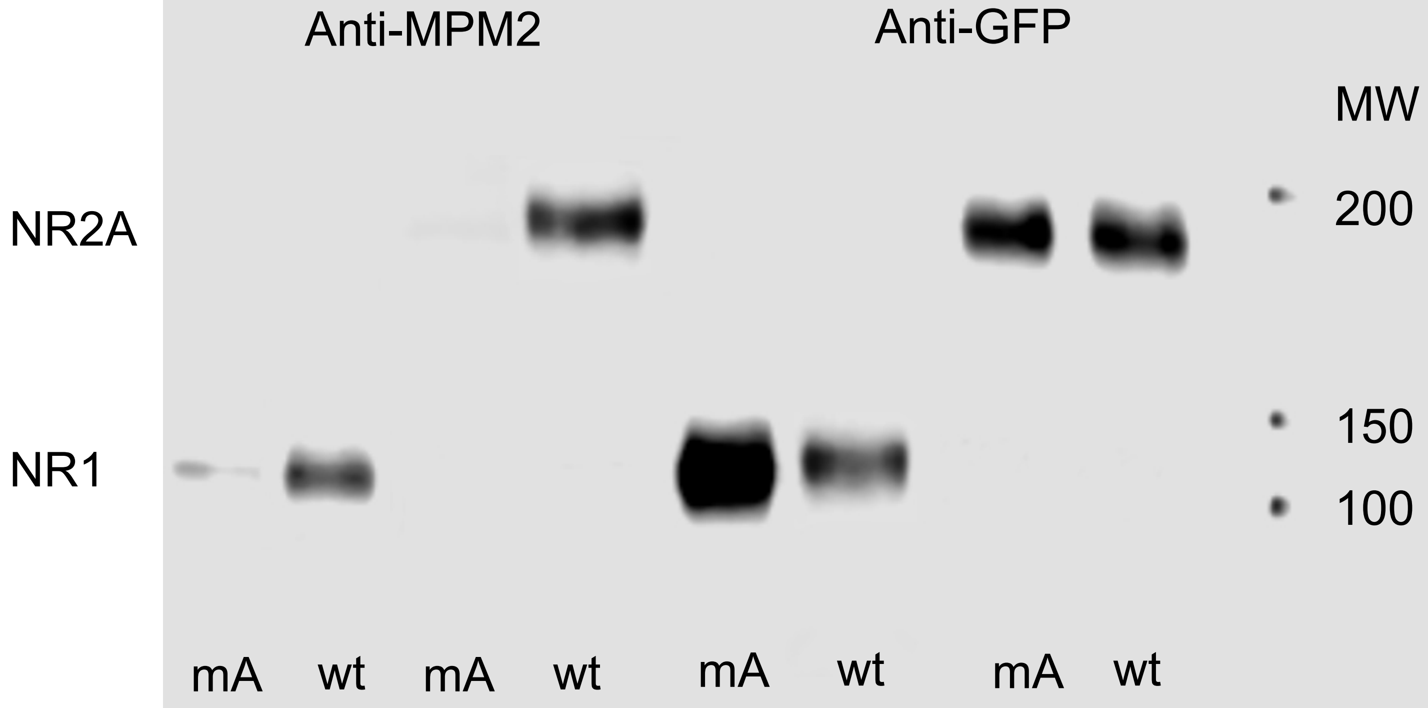

Wild Type and alanine mutant NMDA receptors total vs biotynylated

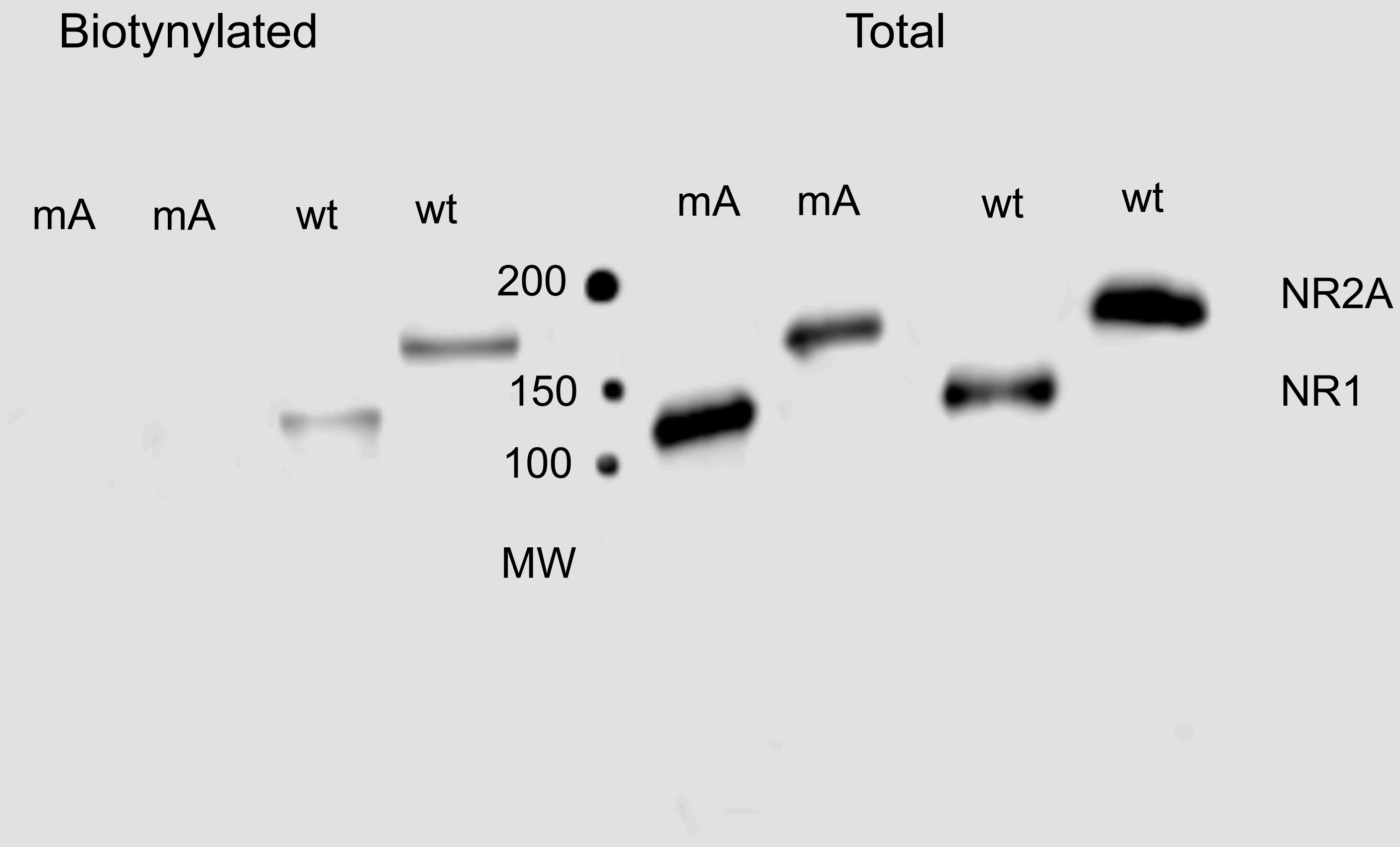

Wild Type and alanine mutant NMDA receptors combinations total vs biotynylated

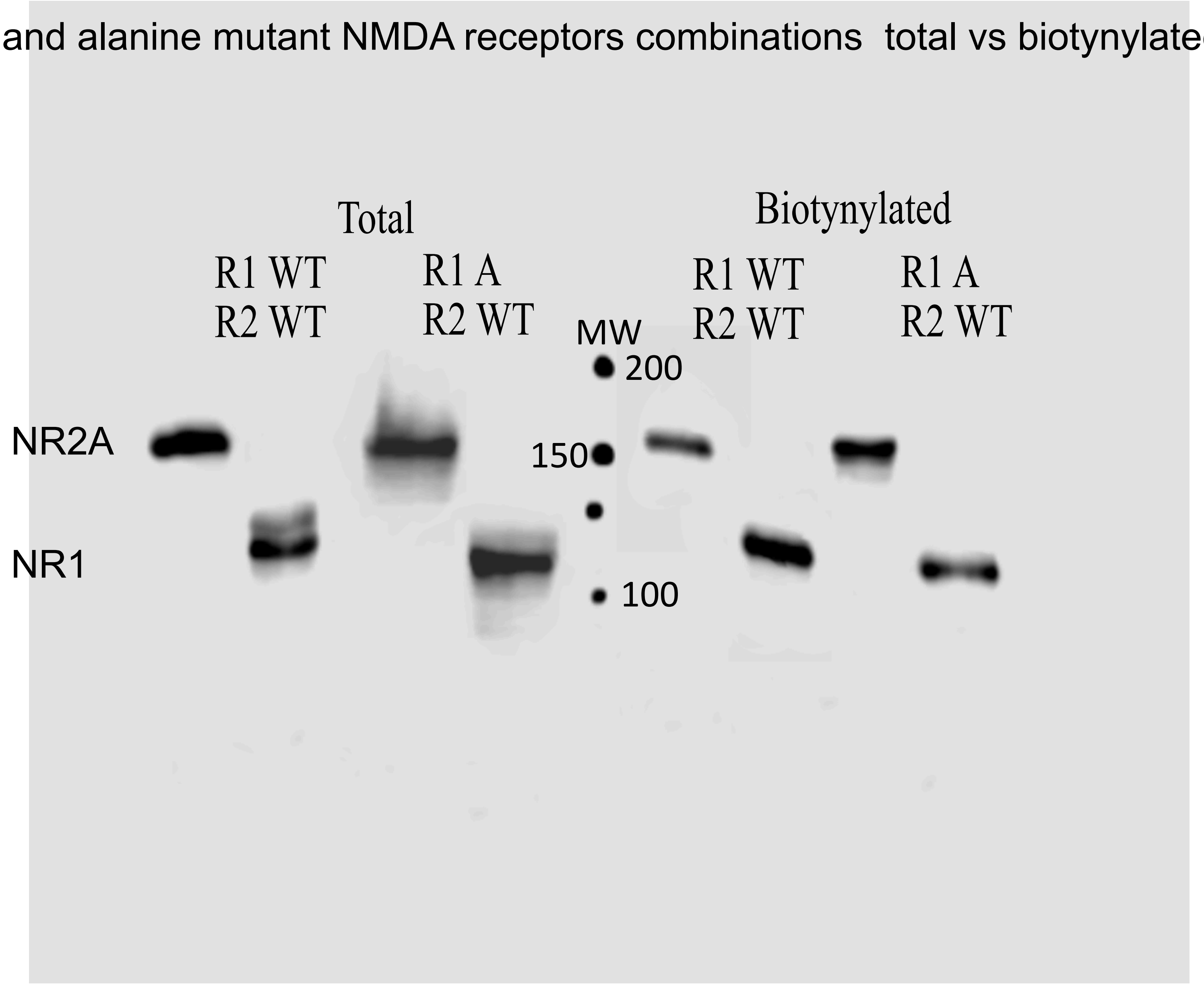

Supplementary figure 2. Full-length western blots. Each blot is self explanatory with the legends included.

In vitro phosphorylation assay full length blots

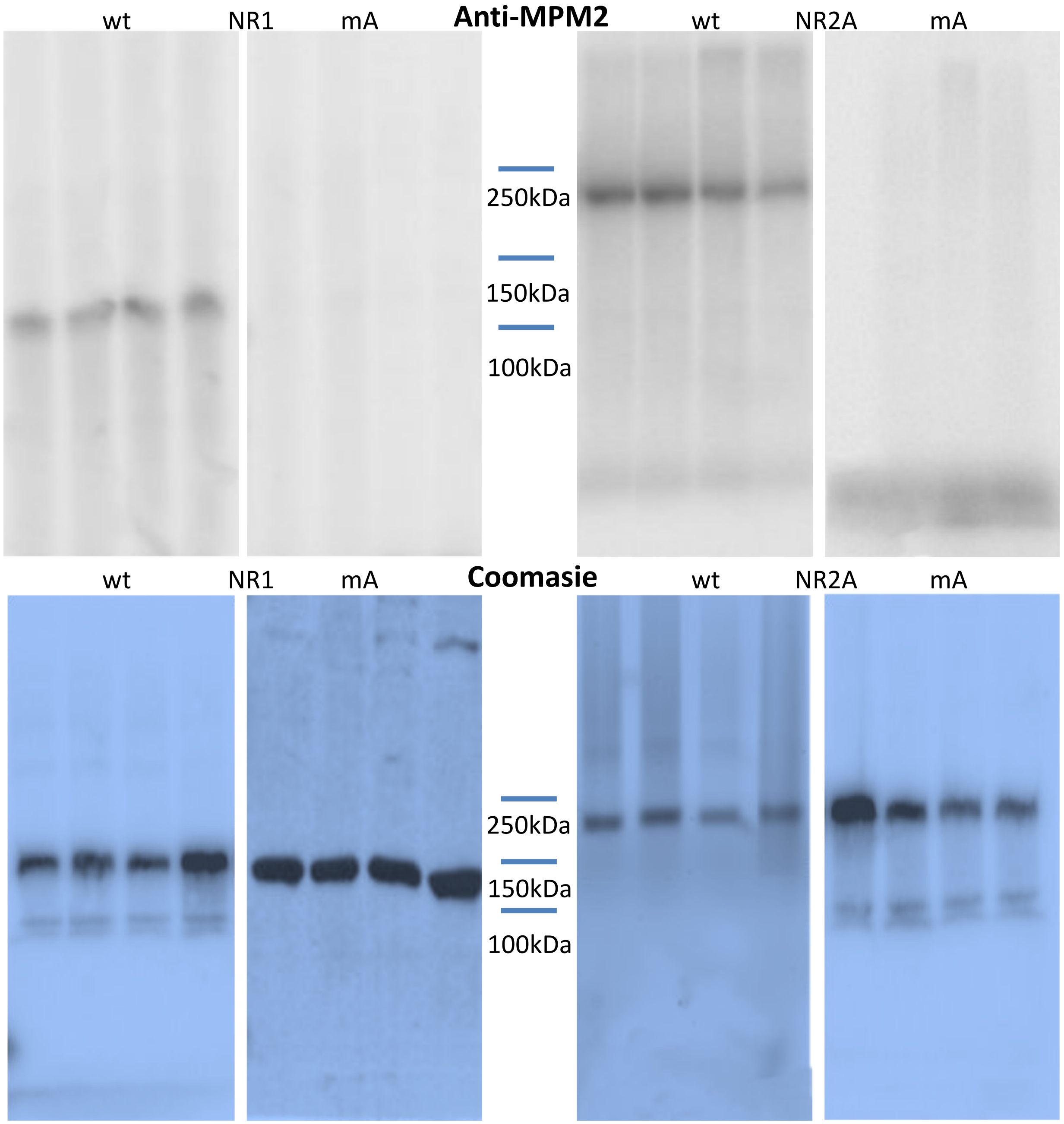

Control NMDARs not incubated with phosphorylation cocktail
Full length bots

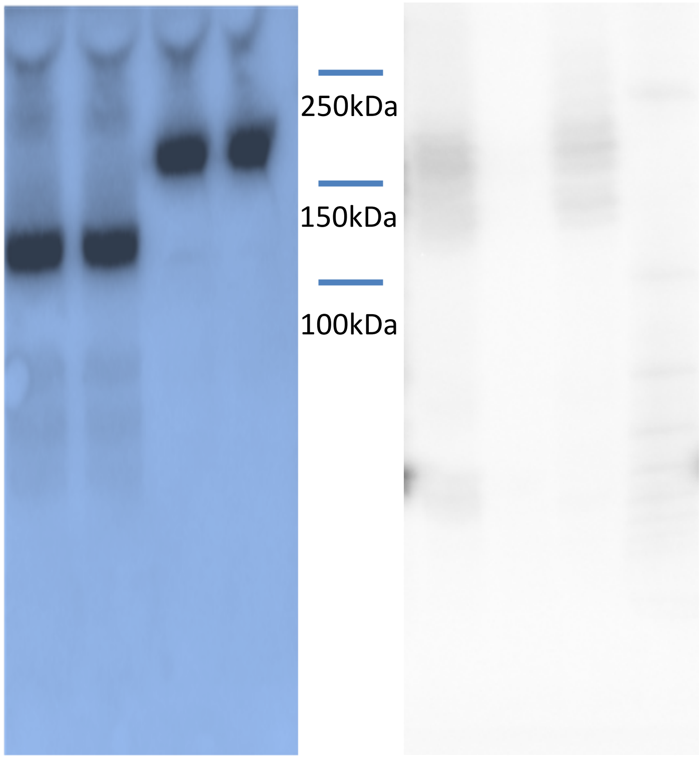

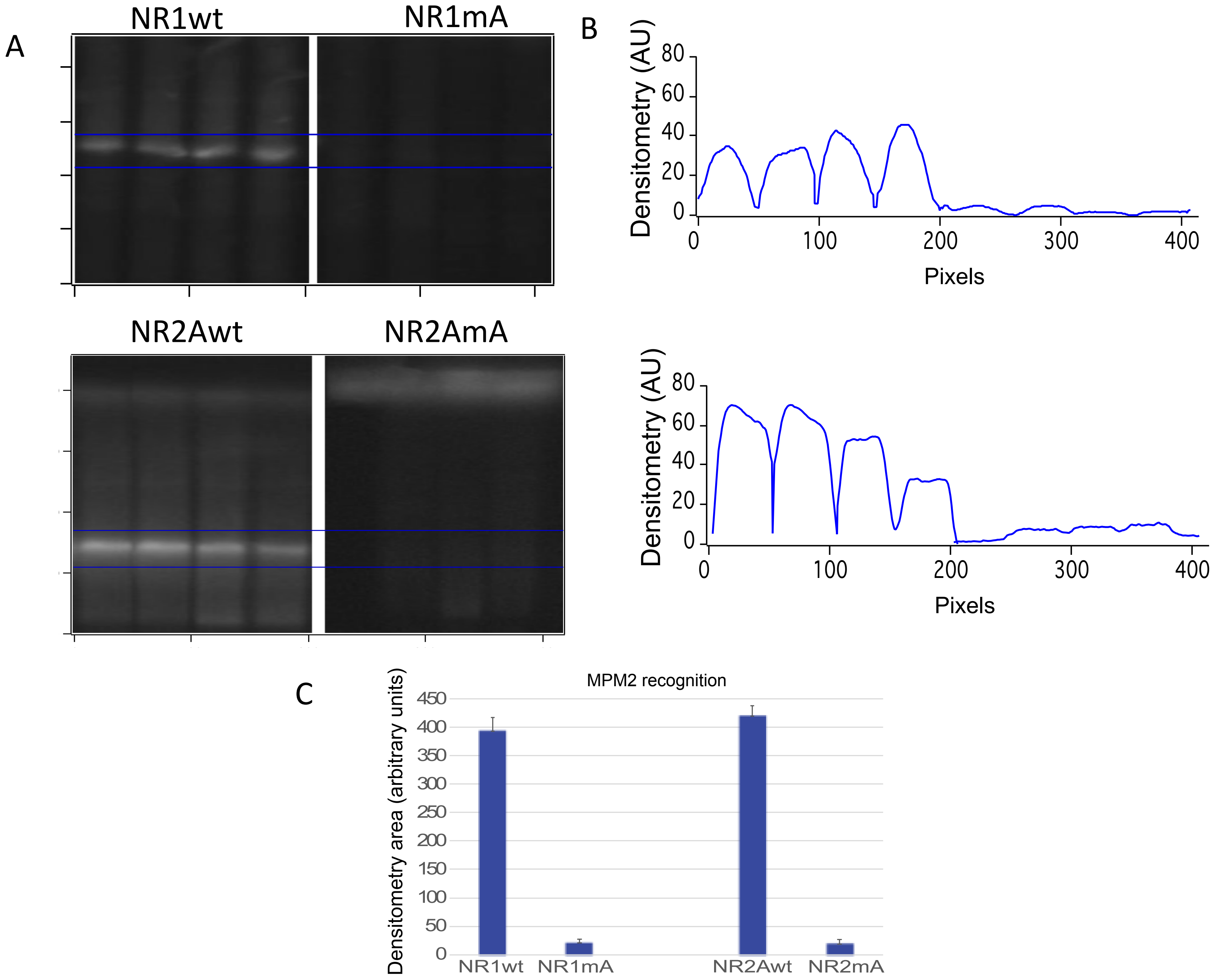

A
NR1wt
NR1mA
NR2Awt
NR2AmA
B
Densitometry (AU)
Pixels
C
MPM2 recognition
Densitometry area (arbitrary units)
NR1wt
NR1mA
NR2Awt
NR2mA

Supplementary figure 3. Densitometry analysis for NMDAR wild type and alanine mutant subunits. A) Western blots showing the recognition by the MPM2 antibody of purified *in vitro* phosphorylated NR1 (upper panels) and NR2A (lower panels). Each lane represent replicas from independent phosphorylation experiments**. B)** Line plot analysis showing the intensity of the bands for each NMDA receptor. Blue lines indicate the area used for the quantification. **C)** Mean +- standard deviation of the area under the curves obtained from B for the 4 replicas under each condition.

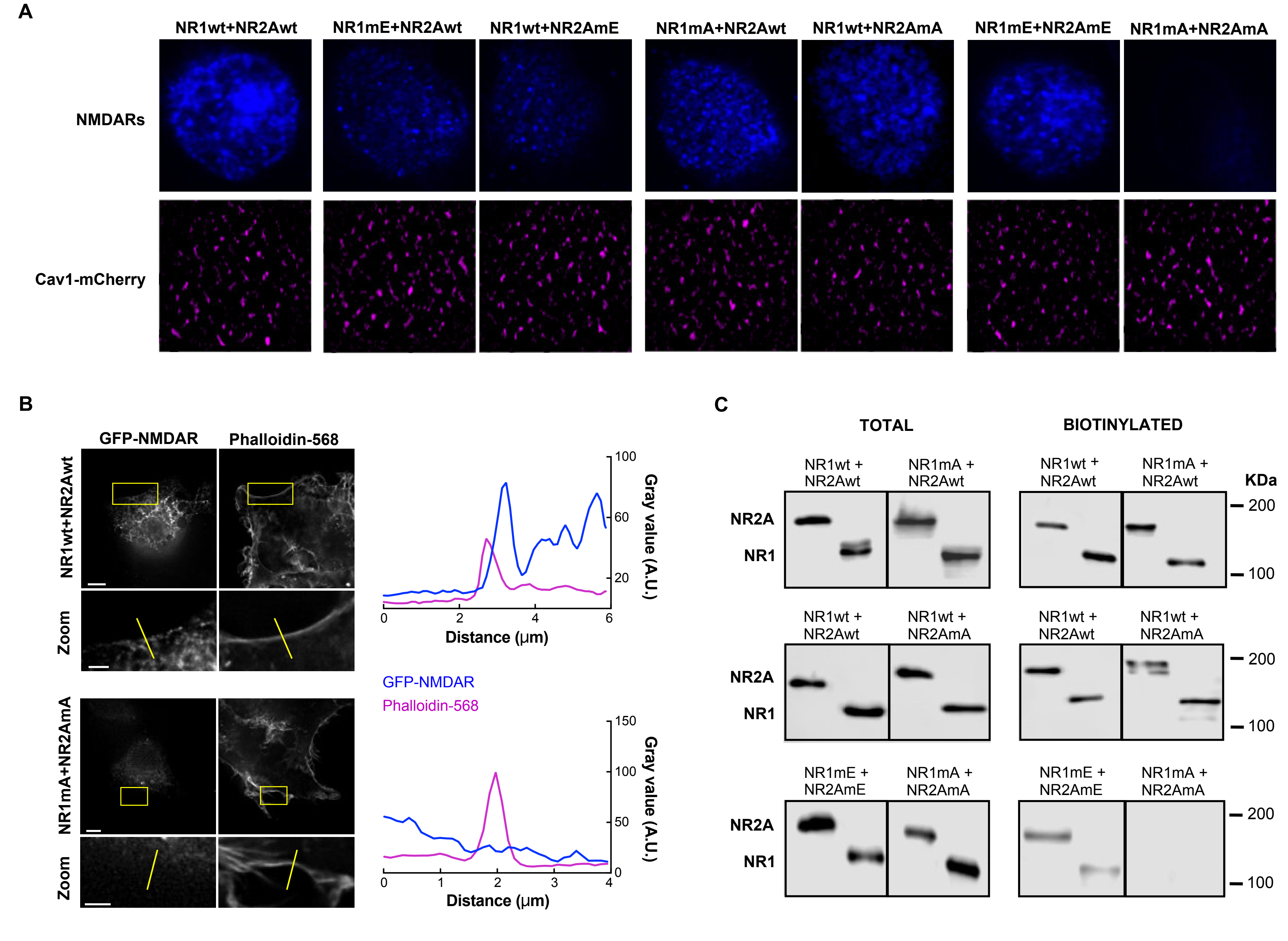

Supp figure 2

Supplementary figure 4. The double alanine mutant of NMDA receptors does not reach the plasma membrane.

A) TIRF images of HEK293 cells transfected with different versions of GFP-NMDARs (letters up) and Caveolin1-mCherry to label the cell membrane. Scale bar: 5 µm. **B)** Widefield deconvolved images of tsa201 cells transfected with different versions of the GFP-NMDARs (left letters) and labeled with Phalloidin-568 to mark the cell membrane. Single focal planes are shown. Plot profiles of the yellow lines marked in the magnifications are shown on the right. Scale bar: 5 µm for original pictures and 2 µm for the magnifications (zoom). **C)** Total amount (left) of different versions of the GFP-NMDARs (letters up the boxes) and amount in plasma membrane (biotinylated, right).
