# Supplementary material for: Phosphorylation of NMDA receptors by cyclin B/CDK1 modulates calcium dynamics and mitosis
Source: Commun Biol. 2020 Nov 12;3:665. doi: 10.1038/s42003-020-01393-3 (PMC7665045; doi:10.1038/s42003-020-01393-3)
Supplement: Supplementary file 2 — Description of Additional Supplementary Files [file 42003_2020_1393_MOESM2_ESM.pdf]

**Description of Additional Supplementary Files**

**File Name:** Supplementary Video 1

**Description:** Time-lapse microscopy of HEK293 cells transfected with NMDAR phosphomimetic mutants show lagging chromosomes and micronuclei. H2B-mCheery was used to follow DNA.

**File Name:** Supplementary Data 1
**Description:** Source Data
